# Combination Treatment with Free Doxorubicin and Inductive Moderate Hyperthermia for Sarcoma Saos-2 Cells

**DOI:** 10.3390/ph18060852

**Published:** 2025-06-06

**Authors:** Valerii E. Orel, Anatolii G. Diedkov, Vasyl V. Ostafiichuk, Sergii A. Lyalkin, Igor O. Tkachenko, Denys L. Kolesnyk, Valerii B. Orel, Olga Yo. Dasyukevich, Oleksandr Yu. Rykhalskyi, Oleksii V. Movchan, Alexander Yu. Galkin, Anna B. Prosvietova

**Affiliations:** 1 National Cancer Institute, 33/43 Zdanovska Str., 03022 Kyiv, Ukraine; 2 National Technical University of Ukraine “Igor Sikorsky Kyiv Polytechnic Institute”, 16/2 Yangel Str., 03056 Kyiv, Ukraine; 3 R.E. Kavetsky Institute of Experimental Pathology, Oncology and Radiobiology, 45 Vasylkivska Str., 03022 Kyiv, Ukraine

**Keywords:** osteosarcoma, free doxorubicin, inductive moderate hyperthermia, antitumor effect, reactive oxygen species, p14^ARF^, epidermal growth factor receptor, tumor cell heterogeneity

## Abstract

**Background:** Osteosarcoma (OS) is the most common primary malignant bone tumor. Doxorubicin (DOX) is extensively used in OS chemotherapy, yet improving patient outcomes remains challenging. This study investigated the effect of free DOX combined with inductive moderate hyperthermia (IMH) on Saos-2 human OS cells. **Methods**: Cell viability was assessed by trypan blue exclusion. Flow cytometry analyzed apoptosis, necrosis, and reactive oxygen species (ROS) in cells exposed to control (no treatment), IMH (42 MHz frequency, 500 μT magnetic field induction, 564 V/m electric field strength, 15 W output power, and 30 min duration) alone, DOX (0.06 μg/mL) alone, or DOX combined with IMH. The expression of p14^ARF^ tumor suppressor and epidermal growth factor receptor (EGFR) was evaluated by immunocytochemistry. Spatial autocorrelation analysis quantified the heterogeneity of p14^ARF^ and EGFR distributions in acquired images. **Results**: The half maximal inhibitory concentration (IC_50_) of DOX in Saos-2 cells had minimal variation between 48 h (0.060 ± 0.01 μg/mL) and 72 h (0.055 ± 0.003 μg/mL). DOX + IMH resulted in a 15% increase in early apoptosis and a 20% elevation in ROS levels compared with DOX alone. Immunocytochemical analysis revealed a 37% increase in p14^ARF^ and a 32% reduction in EGFR expression following combined treatment in comparison to DOX alone. Image analysis showed that DOX + IMH treatment caused the highest Moran’s index values for p14^ARF^ and EGFR, reflecting less heterogeneous spatial distributions (*p* < 0.05). **Conclusions**: IMH enhanced DOX-induced cytotoxicity in Saos-2 cells by initiating ROS-mediated apoptosis and reducing heterogeneity of cellular responses.

## 1. Introduction

Osteosarcoma (OS) is a rare but aggressive form of bone cancer, accounting for approximately 40–50% of bone sarcomas. OS primarily affects adolescents and young adults, with a peak incidence from the ages of 10 to 25. While most cases occur sporadically, a small percentage may be associated with inherited genetic disorders. The development of chromosomal abnormalities in cells from sarcoma specimens has been found to contain oncogenes, e.g., epidermal growth factor receptor (EGFR), and tumor suppressor genes, e.g., p14^ARF^, which contribute to uncontrolled cell growth. Treatment for localized OS, diagnosed in as many as 80% of cases, typically involves a combination of neoadjuvant and adjuvant chemotherapy with surgery. This approach has improved treatment outcomes in patients, with observed cure rates ranging from 60% to 70%. The prognosis is less favorable, however, for patients with metastatic disease or tumors located in the axial skeleton, where cure rates may be as low as 30%. Nevertheless, possible risks and anticipated long-term side effects of given therapy, such as cardiovascular and neurodegenerative disorders and secondary cancers, should be considered since they substantially impact the quality of life for OS survivors [1,2,3].

Doxorubicin (DOX), an anthracycline antibiotic, is widely used as a first-line treatment for bone sarcomas. There are several mechanisms by which DOX acts on cancer cells: DNA intercalation, topoisomerase II inhibition, and free radical formation. It is important to note that DOX-induced free radical generation depends on drug metabolism to semiquinone intermediate [4]. Further involvement of semiquinone DOX in redox cycling gives rise to superoxide and hydrogen peroxide. Anthracyclines also play a role in redox cycling between ferrous and ferric iron ions, disrupting iron regulation in cancer cells and producing reactive oxygen species (ROS) through the Fenton and Haber–Weiss reactions [5,6]. An imbalance between ROS generation and antioxidant capacity leads to oxidative stress. ROS cause substantial damage to DNA, proteins, and lipids in the cells. They are also integrated into redox signaling pathways that regulate cell differentiation, proliferation, and death [7]. DOX can be given as a free drug or nanoparticle formulation encapsulated in liposomes. A number of clinical factors, including patient comorbidities and toxicity from previous chemotherapy courses, guide the appropriate choice of drug form [8]. Compared with liposomal DOX, free DOX is eliminated faster from blood circulation, as it has a shorter half-life and higher total body clearance [9].

Non-ionizing electromagnetic fields have previously been shown to modulate the levels of ROS [10]. Moreover, electromagnetic fields applied within the radiofrequency range can enhance the antitumor efficacy of chemotherapeutic drugs that produce free radicals to inflict damage on cancer cells [11]. The mechanism by which electromagnetic fields modify the levels of free radicals in the cells is based on the conversion between the singlet and the triplet spin states of unpaired electrons in the radical pair, wherein a shift towards the singlet state is more likely to promote ROS generation and oxidative stress [12,13,14,15]. In addition, spin state conversions of ion–radical pairs induced by Zeeman interactions and hyperfine coupling under the influence of an applied field contribute to biochemical processes, such as the enzymatic synthesis of ATP [16]. Exposure of cancer cells to electromagnetic fields also affects gene transcription and protein expression. Yet, the parameters of applied fields, such as frequency, amplitude and waveform, can be tailored to target specific protein interactions [17].

One approach to translating the biological effects of non-ionizing electromagnetic radiation into clinical practice is inductive moderate hyperthermia (IMH). In this treatment modality, the source of heating comes from eddy currents induced in tumor tissue in response to an applied electromagnetic field. Radiofrequency fields within the megahertz range manipulate ionic movement, giving rise to conduction current flows, and, to a lesser extent, cause dipolar molecules to oscillate [18]. The main advantages of IMH over other hyperthermia methods include deeper tissue penetration, localized heating, and compatibility with a range of tumor locations. Unlike high-temperature ablation techniques, IMH is intended to activate apoptotic signaling cascades through ROS generation rather than to cause necrotic cell death triggered by heat stress [19]. This minimizes collateral damage from the inflammatory response to the surrounding tissues, which in turn increases the proportion of patients undergoing organ-conserving surgery and improves 5-year overall survival [11].

IMH exploits both the thermal (moderate heating ≤ 42 °C) and the non-thermal (ROS and ion transport modulation) effects of radiofrequency electromagnetic fields, thereby providing a rationale for combining IMH with chemotherapy to produce a synergistic effect via enhanced oxidative stress [20,21,22,23]. Prior work reported more favorable outcomes of chemotherapy combinations with regional hyperthermia for patients with soft-tissue sarcoma than chemotherapy alone [24,25]. Synergistic effects of IMH and DOX are largely attributed to enhanced tumor perfusion, improved drug delivery, and ROS-mediated sensitization of cancer cells [26,27]. Nevertheless, the role of IMH in increasing the antitumor efficacy of free DOX in OS cells has not been well studied. Herein, we aim to evaluate the effects of free DOX in combination with IMH on Saos-2 OS cells. The data obtained are compared with our previous in vitro study focused on liposomal DOX [28].

## 2. Results

### 2.1. Cytotoxic Response

**Figure 1:**
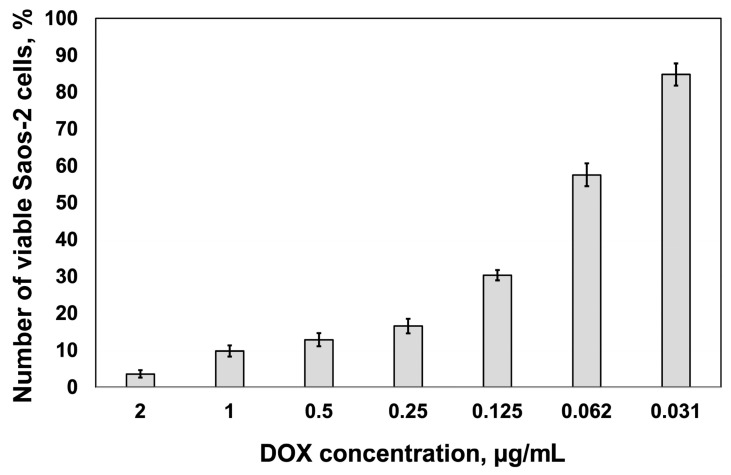
Cytotoxicity IC_50_ of Saos-2 cell line exposed to DOX for 48 h.

The half maximal inhibitory concentration (IC_50_) values for DOX treatment in the Saos-2 cell line at 48 h of incubation are shown in Figure 1. There was a small difference in IC_50_ between 48 h (0.060 ± 0.01 μg/mL) and 72 h (0.055 ± 0.003 μg/mL) of incubation with the drug, which is consistent with the results of previous work in Ref. [29]. This observation indicates that Saos-2 cell responses to DOX were relatively stable over the given duration of drug exposure.

### 2.2. Apoptosis and Necrosis Detection

**Figure 2:**
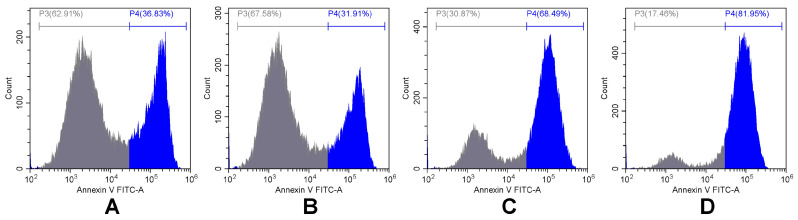
Representative flow cytometry analysis data from annexin V assay in Saos-2 cells: control (**A**), IMH (**B**), DOX (**C**), and DOX + IMH (**D**). P3 includes cells stained negatively for annexin V; P4 includes cells stained positively for annexin V.

**Figure 3:**
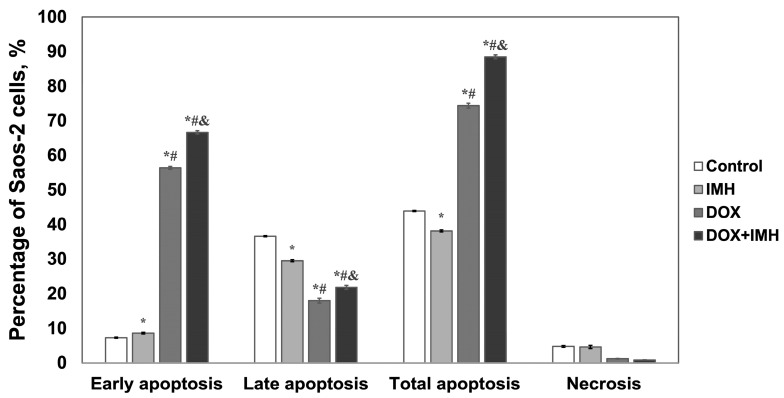
Flow cytometry analysis of apoptosis and necrosis in Saos-2 cells. * Significant difference from control; ^#^ significant difference from IMH; ^&^ significant difference from DOX (*p* < 0.05).

In our analysis, we discriminated between early apoptotic, late apoptotic, and necrotic cell populations by gating them in four regions on propidium iodide conjugated with energy-coupled dye (PI-ECD) versus annexin V conjugated with fluorescein (annexin V-FITC) dot plots, as described in our previous work [28]. Cell debris and doublets were excluded from the analysis. Treatment-induced changes in Saos-2 cell death profiles are illustrated in representative histograms comparing the distribution of annexin V-FITC (Figure 2) and quantified as the percentages of cells undergoing apoptosis and necrosis (Figure 3) at 48 h after exposure to no treatment, IMH, DOX, or DOX + IMH.

Exposure to DOX alone and DOX + IMH resulted in a 7.7- and 9.1-fold increase in the percentage of early apoptotic Saos-2 cells compared with control cells given no treatment. The percentage of cells undergoing early apoptosis was 15% higher in response to IMH than in the control group. By comparing the effects of DOX and DOX + IMH, we found that the exposure of cells to IMH induced an additional 15% increase in the early apoptotic fraction compared to DOX treatment alone. A lower percentage of Saos-2 cells in the late apoptosis stage was likely observed due to competition between DNA intercalation of DOX and propidium iodide. Nonetheless, the combined action of DOX with IMH led to the highest fraction of cells undergoing apoptosis in total (*p* < 0.05). There was no significant difference in the percentage of necrotic Saos-2 cells between the groups.

We next sought to study the effects of DOX and IMH on ROS generation involved in apoptosis and necrosis [30].

### 2.3. Reactive Oxygen Species Measurements

**Figure 4:**
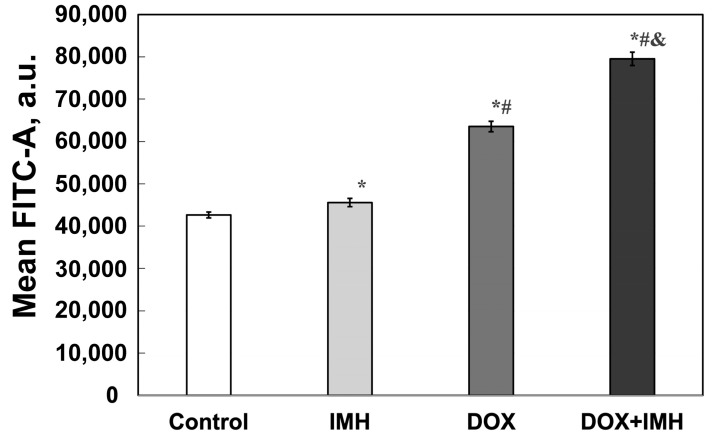
Flow cytometry measurement of ROS produced in Saos-2 cells. * Significant difference from control; ^#^ significant difference from IMH; ^&^ significant difference from DOX (*p* < 0.05).

As shown in Figure 4, either IMH or DOX action alone increased the level of ROS produced in Saos-2 cells by 7% and 33% compared with the control group. The combination treatment with DOX and IMH, however, led to a 47% elevation in ROS level in comparison with untreated cells, a 20% rise compared with DOX alone and a 43% increase compared with IMH exposure (*p* < 0.05). It is known that ROS are involved in numerous signal transduction pathways in cancer cells, including p14^ARF^ tumor suppressor and EGFR [31,32].

### 2.4. Expression of p14^ARF^ and Epidermal Growth Factor Receptor

The effects of either DOX or IMH alone and DOX combination with IMH on p14^ARF^ and EGFR expression were compared to study the molecular mechanisms by which electromagnetic fields may predispose Saos-2 cells to apoptosis and necrosis. Prior studies have provided evidence that the tumor suppressor protein p14^ARF^ medi-ates apoptosis in OS cell lines lacking functional p53, namely, Saos-2 cells [33]. Despite their mesenchymal origin, Saos-2 cells express EGFR on the cytoplasmic membrane surface, where the receptor serves as a signal transducer to activate molecular path-ways, leading to DOX resistance and cell proliferation [34,35].

**Figure 5:**
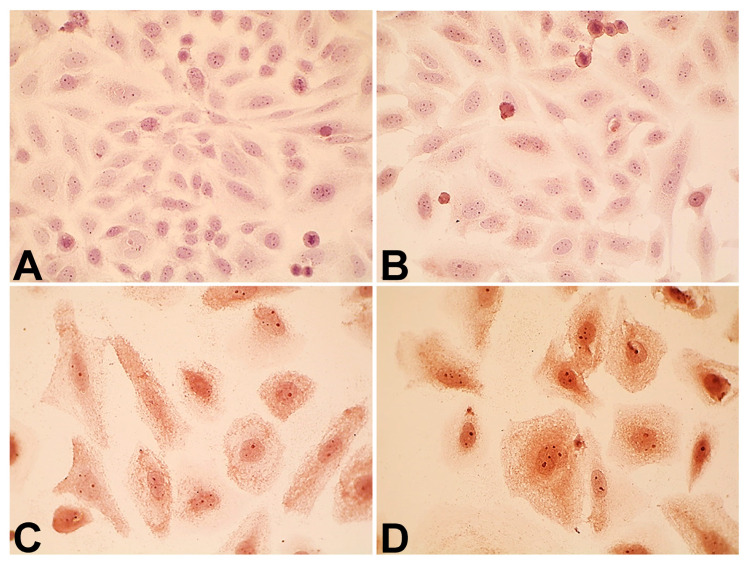
Immunocytochemical staining of p14^ARF^ in Saos-2 cells (magnification 1000×): control (**A**), IMH (**B**), DOX (**C**), and DOX + IMH (**D**).

**Figure 6:**
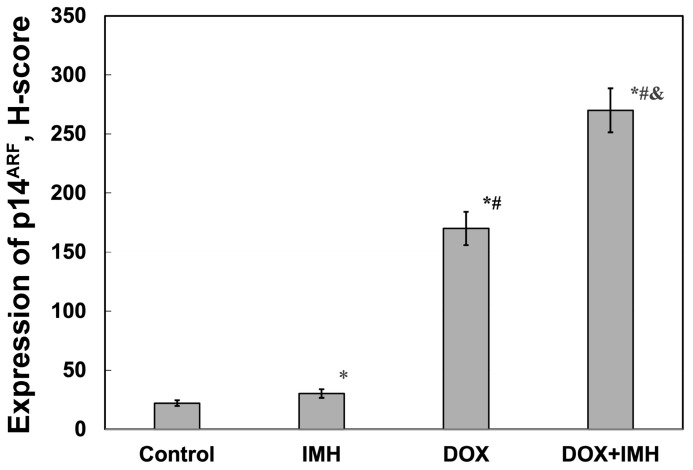
Expression of p14^ARF^ protein in Saos-2 cells. * Significant difference from control; ^#^ significant difference from IMH; ^&^ significant difference from DOX (*p* < 0.05).

IMH caused a 27% increase in the level of p14^ARF^, whereas DOX displayed an 87% higher level of tumor suppressor than Saos-2 cells in the control group (Figure 5 and Figure 6). DOX combination with IMH further increased the level of p14^ARF^ expression by 92% and 37% compared with the untreated cells and DOX alone, respectively (*p* < 0.05).

**Figure 7:**
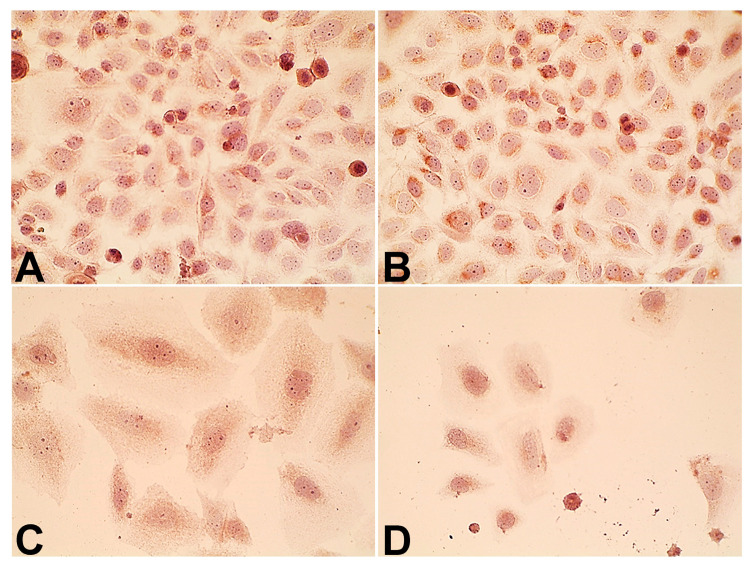
Immunocytochemical staining of EGFR in Saos-2 cells (magnification 1000×): control (**A**), IMH (**B**), DOX (**C**), and DOX + IMH (**D**).

**Figure 8:**
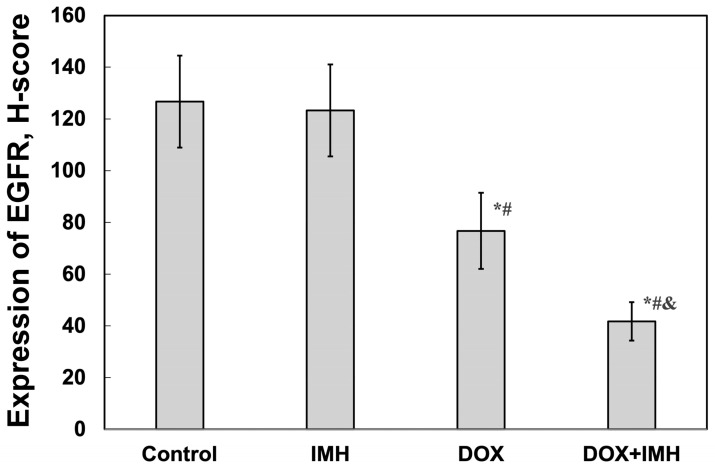
Expression of EGFR in Saos-2 cells. * Significant difference from control; ^#^ significant difference from IMH; ^&^ significant difference from DOX (*p* < 0.05).

We did not observe a significant difference in the level of EGFR between Saos-2 cells subjected to no treatment and IMH. However, DOX exposure decreased the EGFR level by 40% compared with the control group (Figure 7 and Figure 8). The exposure of Saos-2 cells to DOX and IMH combination resulted in a 67% and 32% decrease in the EGFR level compared with control and DOX-treated cells, respectively (*p* < 0.05). These findings suggest that DOX and IMH have a synergistic effect on p14^ARF^ and EGFR expression in Saos-2 cells.

**Figure 9:**
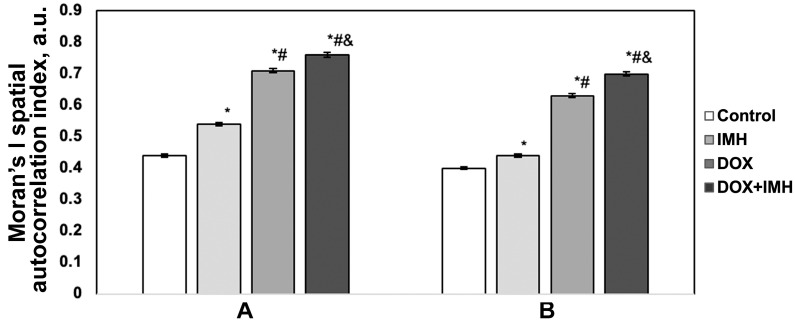
Spatial autocorrelation analysis of p14^ARF^ (**A**) and EGFR (**B**) in Saos-2 cells. * Significant difference from control; ^#^ significant difference from IMH; ^&^ significant difference from DOX (*p* < 0.05).

Figure 9 shows the results of spatial autocorrelation analysis based on Moran’s index (Moran’s I) calculated for p14^ARF^- and EGFR-stained Saos-2 cell immunocytochemistry images. DOX and IMH combination resulted in the highest value of Moran’s I, indicating the lowest degree of spatial heterogeneity for p14^ARF^ and EGFR distributions compared with no treatment, IMH, and DOX alone. In addition, exposure to IMH also led to a statistically significant increase in Moran’s I compared with the control group (*p* < 0.05).

## 3. Discussion

Improving cure rates in OS patients still represents a significant challenge. DOX is one of the most useful chemotherapeutic drugs in neoadjuvant and adjuvant treatment regimens [36]. Tissue hypoxia is a major source of chemo- and radiotherapy resistance of bone tumors. While bone is considered a highly vascularized connective tissue, with increased oxygen demand during growth and repair, its perfusion shows a heterogeneous pattern, with partial oxygen pressure ranging from 50 mm Hg (normoxic region) in the periosteum to 9.9 mm Hg (hypoxic region) in the extravascular bone marrow compartment [37]. The bone microenvironment is hypoxic given the low oxygen tension in the sinusoids, high oxygen consumption by haemopoietic cells, and increased resistance to oxygen diffusion in the bone matrix [38,39]. ROS can influence the relationship between osteoblasts and osteoclasts, leading to decreased mineralization, increased osteoblast apoptosis, and increased bone resorption under hypoxic conditions [40]. Among other osteoblast-like features, Saos-2 cells are capable of secreting extracellular matrix, which makes it possible to use them as an in vitro model of OS. Saos-2 cells were found to reach a stage similar to mature human osteocytes in response to different oxygen concentrations [41]. Furthermore, prior work has already established that non-ionizing electromagnetic fields enhance the antitumor activity of DOX in cancer cells by inducing changes in the respiratory chain of mitochondria and subsequent ROS-mediated cell damage [42]. In the present study, we exposed Saos-2 cells to radiofrequency electromagnetic fields in order to examine the effects of IMH and free DOX on cell viability, ROS generation, and p14^ARF^ and EGFR expression.

There are two primary mechanisms by which DOX combined with IMH could initiate a more pronounced antitumor effect in Saos-2 cells than DOX alone: (1) local temperature increase, and (2) a change in electron transfer processes and spin dynamics of radical pairs. While DOX undergoes redox cycling and acts as a pro-oxidant, IMH facilitates DOX-induced ROS generation, contributing to enhanced oxidative stress in cancer cells. This synergistic action increases mitochondrial membrane permeability, leading to the release of cytochrome c and activation of intrinsic apoptotic pathways. At the same time, elevated ROS levels can irreversibly damage lipids, proteins, and nucleic acids, disrupting redox-dependent signaling pathways in the cells [43,44,45]. In the following paragraphs, we discuss the relevance of our results to IMH effects on DOX-induced ROS generation and p14^ARF^ and EGFR expression, as well as Saos-2 cell apoptosis. We also compare the obtained results with our previous investigation focused on the application of IMH to potentiate the action of liposomal DOX.

Encapsulating DOX in liposomes increased the IC_50_ in Saos-2 cells from 0.06 ± 0.01 μg/mL (free DOX) to 1.8 ± 0.2 μg/mL (liposomal DOX) at 48 h incubation. From 48 to 72 h, the IC_50_ values for liposomal DOX showed a 4.7-fold decrease (*p* < 0.05) [28], while there was no significant difference between cells subjected to free DOX. Likewise, previous studies noted a more pronounced cytotoxic effect of free DOX than its liposomal formulation [46]. The DOX + IMH combination resulted in a significantly larger fraction of apoptotic cells (Figure 3) and a higher level of ROS (Figure 4) produced in Saos-2 cells than either DOX or IMH alone, as determined by using flow cytometry (*p* < 0.05). IMH and DOX initiated apoptosis as a prevalent form of cell death rather than necrosis. These findings are consistent with those reported for liposomal DOX and IMH. Together with the work described above, our results demonstrate that free DOX combined with IMH led to a 19% increase in Saos-2 cells undergoing early apoptosis and a 29% elevation in ROS level compared with the combination treatment based on liposomal DOX (*p* < 0.05).

As is well known, DOX causes mitochondrial iron overload through redox reactions between Fe²⁺ and Fe³⁺ ions, wherein ROS arise as byproducts of the Fenton and Haber–Weiss reactions. Iron homeostasis appears to play an important role in tumor cells, contributing to redox signaling, ROS generation, oxidative stress, cell damage, and death [47]. This is one of the mechanisms through which DOX inhibits cancer cell proliferation in addition to DNA intercalation and topoisomerase-2A inhibition [48]. Since iron ions have unpaired electrons in their outer shell, they exhibit magnetic properties, allowing external electromagnetic fields to interact with them and induce both thermal and non-thermal effects. The absorption of electromagnetic energy in biological objects is associated with local temperature rise—for instance, due to viscous resistance to rotating magnetic particles under the influence of an applied field (Brownian relaxation). Note that temperatures above 42 °C are generally avoided during IMH to minimize the risk of side effects associated with chemotherapy resistance mediated through heat-shock protein expression, tumor vascular shutdown, and heat pain thresholds. On the other hand, non-thermal effects of electromagnetic fields, such as magnetochemical and magneto-mechanical effects, are based on transitions between different electronic spin states of the radical pair and the magnetic force exerted on magnetic particles in response to the applied field, respectively. The conversion of the triplet state to the singlet state increases the probability of free radical recombination and may also promote ROS formation [21,22,47,49,50].

In contrast to free DOX, liposomal formulations are composed of an aqueous core with DOX enclosed within a phospholipid bilayer containing unsaturated lipids [51]. It becomes immediately apparent that unsaturated linkages in these lipids are susceptible to peroxidation initiated by ROS. More importantly, lipid peroxidation can alter the anticancer activity of DOX released in the tumor and its microenvironment. The proposed mechanism for free and liposomal DOX involvement in free radical reactions is presented in Equations (1)–(8) based on Refs. [28,42,46,47,48,51,52,53]. As shown in Equation (1), DOX engages in redox cycling between Fe^2+^ and Fe^3+^, leading to hydroxyl radical (^•^OH) formation. DOX can catalyze the transfer of electrons from Fe^2+^ to molecular oxygen, giving rise to superoxide anion (O_2_^•−^) radical in Equation (2). The conversion of superoxide radical to hydrogen peroxide (H_2_O_2_) by the enzyme superoxide dismutase in Equation (3) further contributes to the Fenton reaction. Hydroxyl radical in Equation (4) initiates lipid peroxidation chain reactions in the cell membranes, including Equation (5). Unsaturated fatty acids within the phospholipid (LH) bilayer of DOX-containing liposomes are also susceptible to ROS oxidation, resulting in lipid hydroperoxide (LOOH) formation, shown in Equation (6), which in turn destabilizes the liposome and promotes DOX release. The application of electromagnetic fields to induce moderate heating of Saos-2 cells also influences electron transfer processes in the cells and medium through magnetochemical effects, favoring an increase in DOX semiquinone (DOX^•−^) radical in Equation (7) and Fe^2+^ concentration in Equation (8). The latter facilitates the Fenton and Haber–Weiss reactions involved in oxidative stress.

**Free DOX:**

Fe^2+^ + H_2_O_2_ → Fe^3+^ + HO^−^ + ^•^OH (1)

O_2_ + e^−^ → O_2_^•−^ (2)

2O_2_^•−^ + 2H^+^ → H_2_O_2_ + O_2_ (3)

RH + ^•^OH → R^•^ + H_2_O (4)

R^•^ + O_2_ → RO_2_^•^ (5)

**Liposomal DOX:**

RO_2_^•^ + LH → LOOH + R^•^ (6)

**IMH:**

(7) $$\mathrm{DOX}+e^{-}\;\overset{electromagnetic\;field}{\to }\;{\mathrm{DOX}}^{•-}$$

(8) $${\mathrm{Fe}}^{3+}+e^{-}\;\overset{electromagnetic\;field}{\to }\;{\mathrm{Fe}}^{2+}$$

Equations (1)–(8) DOX and IMH effects on ROS formation in cancer cells.

A growing body of evidence suggests that ROS serve a critical role in cell signaling pathways, wherein the signal is transduced across a group of proteins via electron transfer processes between individual protein members, forming tightly regulated redox chains [54]. Clearly, local temperature changes also affect electron transport and redox homeostasis in biological objects. It was previously shown that the Fenton and Haber–Weiss reactions are temperature-dependent [55]. For this reason, we applied only a moderate temperature increase (<42 °C) during IMH to prevent hydrogen peroxide from degrading into oxygen and water. Otherwise, higher temperatures would have limited these reactions.

In particular, we found that DOX and IMH increased p14^ARF^ expression in Saos-2 cells (Figure 5 and Figure 6). The upregulation of p14^ARF^ initiates cell cycle arrest and apoptosis following p53-dependent and independent pathways, both of which enhance the efficacy of DOX [56]. Tumor cell responses to oxidative and heat stresses are also mediated by p14^ARF^ through interactions with heat-shock proteins, namely, Hsp70, leading to β-catenin degradation and subsequent apoptosis [57]. Redox reactions regulate various cellular processes, including activation of membrane receptors and ion transport channels. EGFR is an example of a transmembrane receptor whose activation is also influenced by ROS. OS cells express EGFR, functioning as a prevention of self-destructive mechanisms. Interestingly, it was demonstrated that immunotherapeutic agents targeting EGFR were not useful for generating antitumor effects in OS cells [58]. Another study established that the exposure of tumor cells to radiofrequency electromagnetic radiation decreased EGFR levels and reduced cell viability, possibly through changes in electrostatic interactions and non-covalent binding [59]. As shown in Figure 7 and Figure 8, free DOX and IMH action resulted in the lowest level of EGFR expression in Saos-2 cells.

However, the combination of liposomal DOX and IMH caused the highest level of pro-apoptotic Bax protein among the cells subjected to no treatment, liposomal DOX alone or IMH alone. Based on experiments measuring ROS, we propose that different ROS levels in Saos-2 cells initiate different cell signaling pathways. It should be noted that the exposure of Saos-2 cells to free or liposomal DOX with IMH had the highest Moran’s I values calculated for Bax, p14^ARF^, and EGFR spatial distributions on immunocytochemistry images (*p* < 0.05). The higher the Moran’s I value, the lower the heterogeneity of pixel distributions for biomarkers in acquired immunocytochemistry images [60,61,62].

Altogether, enclosing the drug in a phospholipid bilayer alters the uptake and distribution of DOX within cells compared with its free form and initiates a distinct cascade of signaling events. DOX delivery depends on ionization state and membrane binding [63,64]. Combining free DOX or its liposomal formulation with IMH produces changes in the kinetics of free radical chain reactions (Equations (1)–(8)) and ROS diffusion in cells compared with their separate effects. In our earlier study, liposomal DOX induced apoptosis in Saos-2 cells mediated by Bax upregulation, which reflected a response characterized by a lower ROS level. In the current study, free DOX triggered apoptosis through p14^ARF^ upregulation and EGFR downregulation associated with a higher ROS level. These observations are in agreement with the fact that free DOX results in a more pronounced nuclear stress than its liposomal formulation [65]. In addition, we found differences in the spatial heterogeneity of apoptosis-related biomarkers between the two forms of DOX and their combinations with IMH, indicating distinct structural and functional alterations in Saos-2 cell populations. Considering the inherent heterogeneity of cell populations within OS, it might be interesting to evaluate the effects of DOX combined with IMH in a panel of DOX-resistant OS cell lines, such as 143-B-DX-R and Saos-2-DX-R.

Although a wide range of experimental evidence now offers comparisons between free and liposomal DOX combinations with other moderate hyperthermia techniques, far less research has focused on the role of IMH in potentiating DOX cytotoxic activity, particularly in OS models, wherein both the magnetic and the electric components of the applied electromagnetic field contribute to the antitumor effect. A comparative examination of our experimental data, therefore, shows that IMH not only enhances cytotoxic effects initiated by both forms of DOX but also alters the expression and heterogeneity of different cellular responses across Saos-2 cell populations, providing a novel scientific perspective on the inherent heterogeneity of cell populations within osteosarcoma. Moreover, future research is needed to validate our findings in animal models of OS, which will provide a deeper understanding of thermal and nonthermal effects produced by IMH in vivo and potentially accelerate its translation into clinical practice [66,67].

## 4. Materials and Methods

To ensure consistency in comparisons, this paper follows a similar structure and methodology as our earlier work [28]. In the current study, we focus on the cytotoxic effects and underlying mechanisms of free DOX with IMH. Furthermore, we provide a comparative analysis of free and liposomal DOX under identical conditions.

### 4.1. Cell Culture

The Saos-2 cell line is an extensively studied culture model of primary bone tumors derived from the bone of an 11-year-old female patient with OS, which displays epithelial morphology and osteoblastic features, including the ability to synthesize alkaline phosphatase and high resistance to DOX [68]. Saos-2 cells (ATCC HTB-85) were obtained from the cell line bank of R.E. Kavetsky Institute of Experimental Pathology, Oncology and Radiobiology, and cultured in Dulbecco’s modified Eagle’s medium (DMEM/F12, Sigma-Aldrich, Taufkirchen, Germany) supplemented with 10% fetal bovine serum (Sigma-Aldrich, Taufkirchen, Germany) and antibiotic-antimycotic (Sigma-Aldrich, St. Louis, MO, USA) under standard conditions of 37 °C in 5% CO_2_ [69].

### 4.2. Inductive Moderate Hyperthermia

**Figure 10:**
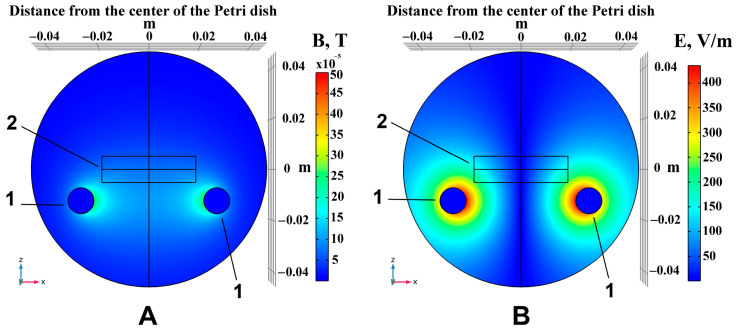
COMSOL simulations of IMH: distribution of the magnetic (**A**) and electric (**B**) fields. 1—Applicator; 2—Petri dish with Saos-2 cells and the medium.

Saos-2 cells were exposed to non-ionizing electromagnetic radiation using a MagTherm device (Radmir, Kharkiv, Ukraine) operated at a 42 MHz frequency and 15 W of output power for 30 min. Exposure plans were prepared using COMSOL Multiphysics v. 5.6 software (COMSOL AB, Stockholm, Sweden) to ensure optimal distribution of electromagnetic fields and temperature. The software provided an interface to couple the magnetic fields and bioheat transfer modules. As the applied field had two components, we created a treatment plan for the magnetic (Figure 10A) and the electric (Figure 10B) field distributions in response to IMH. COMSOL simulations predicted the maximum values of magnetic induction of 500 µT, electric field strength of 564 V/m, and specific absorption rate of 10.6 W/kg. Additionally, we recorded temperature measurements in the center of the Petri dish using a fiber-optic sensor connected to a digital thermometer TM-4 (Radmir, Ukraine) throughout IMH. Saos-2 cells were gradually heated, reaching a temperature of 41.23 °C. A detailed description of IMH planning and methodology can be found in our earlier work [28].

### 4.3. Cell Viability

When Saos-2 cells were 90% confluent, they were harvested using trypsin in saline disodium ethylenediaminetetraacetic acid (S-EDTA) and added to each well of a 96-well plate (TPP, Trasadingen, Switzerland) in DMEM/F12 medium supplemented with 10% fetal bovine serum and antibiotic–antimycotic at a concentration of 10^4^ cells per well. Cells were treated with different concentrations of DOX (Ebewe Pharma, Unterach, Austria, Ministry of Health of Ukraine approval registry: LH8929). The drug was initially supplied in a liquid form as a concentrate (2 mg/mL) for the solution for infusion, then diluted in culture medium and incubated under standard conditions (37 °C, 5% CO_2_) for 48 or 72 h. After incubation with DOX, Saos-2 cells were fixed by adding 10% trichloroacetic acid (Sigma-Aldrich, Taufkirchen, Germany) and stained with sulforhodamine B (Sigma-Aldrich, St. Louis, MO, USA). Excess dye was removed by washing the cells with 1% acetic acid solution (Sigma-Aldrich, Taufkirchen, Germany), and the remaining dye was resolubilized in 10 mM Tris base solution (Sigma-Aldrich, Taufkirchen, Germany) [70]. Sample absorbance at 510 nm was measured by a multi-well spectrophotometer (ThermoLabsystems Multiskan EX, Waltham, MA, USA). IC_50_ values were calculated by linear regression analysis.

The viable Saos-2 cell number was assessed by trypan blue exclusion assay (Sigma-Aldrich, Taufkirchen, Germany) in response to no treatment (control group), IMH exposure, DOX, and DOX combined with IMH [71]. Cells were seeded in Petri dishes with DMEM/F12, 10% fetal bovine serum, and antibiotic–antimycotic at a concentration of 1.5 × 10^5^ per dish. DOX was added at a concentration of 0.06 μg/mL and then the cells were exposed to IMH for 30 min. Following IMH treatment, the Saos-2 cells were incubated under standard conditions for 48–72 h and counted using a hemocytometer (Micromed, Kyiv, Ukraine).

### 4.4. Flow Cytometry

After 48 h of incubation, Saos-2 cells were labeled fluorescently to detect apoptotic and necrotic cells by adding annexin V-FITC and PI-ECD (Dojindo, Munich, Germany) [72,73]. A DxFlex flow cytometer (Beckman Coulter, Brea, CA, USA) was used to examine the resulting samples, analyzing at least 21,000 events for each sample. Data collection and analysis were conducted with the CytExpert v. 2.5 software supplied with the cytometer. The levels of ROS were measured by a 2′,7′-dichlorodihydrofluorescein diacetate (DCFH-DA, Dojindo, Germany) assay according to the methodology described in Ref. [74].

### 4.5. Immunocytochemical Assay

After 72 h of incubation, Saos-2 cells were fixed in methanol (Sigma-Aldrich, USA) and acetone (Chimreserv, Kyiv, Ukraine). Next, primary antibodies recognizing EGFR (clone 111.6, Thermo Scientific, Waltham, MA, USA) and tumor suppressor p14^ARF^ (clone 14P02, Thermo Scientific) were added for 1 h at room temperature [75,76]. Immunodetection was performed using a Super Picture polymer detection kit (Thermo Fisher Scientific) with hematoxylin counterstain. Immunocytochemistry results for these two biomarkers were assessed using the H-score [77].

### 4.6. Image Analysis

Saos-2 cells were segmented in collected immunocytochemistry images by adopting the k-means clustering approach using ImageJ v. 1.53k software (NIH, Bethesda, MD, USA). We then calculated Moran’s I to quantify changes in the spatial heterogeneity of intracellular p14^ARF^ and EGFR distributions using Autocorrelation v. 1.0 software (NCI, Kyiv, Ukraine) [78,79].

### 4.7. Statistical Analysis

Statistical differences were determined using Statistica v. 6.0 (Statsoft Inc., Tulsa, OK, USA) and IBM SPSS Statistics v. 25.0 (IBM Inc., Chicago, IL, USA) software. Two groups were compared using the Student’s *t*-test or the Mann–Whitney U-test. Experimental data between three or more groups were compared using a one-way analysis of variance (ANOVA) followed by the Games–Howell post hoc test or the Kruskal–Wallis test. A *p*-value < 0.05 was taken as statistically significant.

## 5. Conclusions

The objective of this study was to examine the antitumor effect of free DOX combined with IMH on Saos-2 human OS cells, with a particular focus on the interaction of a non-ionizing electromagnetic field with a chemotherapeutic agent acting through the free radical mechanism. In addition, we compared the obtained results with our previously published data on the use of liposomal DOX with IMH.

Here, we show that free DOX combination with IMH caused a 19% increase in Saos-2 cells undergoing early apoptosis and a 29% elevation in ROS compared with the results observed for liposomal DOX with IMH. Using DOX in combination with IMH also resulted in the highest expression of p14^ARF^ tumor suppressor and the lowest expression of EGFR. Immunocytochemistry image analysis revealed the lowest values of Moran’s I for both p14^ARF^ and EGFR, indicating a less heterogeneous pattern of spatial distribution (*p* < 0.05).

In summary, our findings demonstrate that IMH can enhance the antitumor activity of DOX in Saos-2 OS cells, providing experimental data on the more potent effects of IMH combination with free DOX rather than its liposomal formulation. Future preclinical studies are required to compare the efficacy of IMH with free and liposomal DOX in vivo to further support the application of non-ionizing electromagnetic radiation benefits to OS treatment outcomes.

## Data Availability

Data is contained in the paper.

## Abbreviations

The following abbreviations are used in this manuscript:

Annexin V-FITC	Annexin V conjugated with fluorescein
DOX	Doxorubicin
EGFR	Epidermal growth factor receptor
FSC-A/SSC-A	Forward/side scatter area
IC_50_	Half maximal inhibitory concentration
IMH	Inductive moderate hyperthermia
OS	Osteosarcoma
PI-ECD	Propidium iodide conjugated with energy-coupled dye
ROS	Reactive oxygen species
